# Hereditary Angioedema

**DOI:** 10.7759/cureus.42088

**Published:** 2023-07-18

**Authors:** Anjali Banerjee, Natalie Bermudez, Murdoc B Gould, Sidhartha R Ramlatchan, Latha Ganti

**Affiliations:** 1 Emergency Medicine, University of Central Florida, Orlando, USA; 2 Chemistry, Rollins College, Winter Park, USA; 3 Emergency Medicine, Drexel University, Philadelphia, USA; 4 Emergency Medicine, Hospital Corporation of America (HCA) Florida Ocala Hospital, Ocala, USA; 5 Emergency Medicine, Envision Physician Services, Plantation, USA; 6 Emergency Medicine, University of Central Florida College of Medicine, Orlando, USA

**Keywords:** hereditary angioedema, bradykinin, anaphylaxis, c1 esterase inhibitor, heriditary angioedema, angioedema

## Abstract

The authors present the case of a 22-year-old female who reported having a persistent sore throat. The patient had a history of recurring episodes of hereditary angioedema and arrived at the emergency department with her C1-esterase inhibitor. The epidemiology, clinical presentation, and treatment strategies are presented.

## Introduction

Hereditary angioedema (HAE) is a rare but life-threatening autosomal dominant genetic disorder caused by a deficit (type I) or dysfunction (type II) of the C1 esterase inhibitor (C1-INH). The condition results from a mutation in the *SERPING1* gene, which encodes the C1-INH protein resulting in the body's inability to regulate the levels of specific blood plasma proteins, which can lead to episodes of inflammation in various organ systems [1]. There is also a third type (type III) where the C1 esterase level is normal but there is a genetic mutation on the gene that encodes coagulation factor XII. Risk factors for HAE include a family history of the disorder. HAE affects one in 10,000 to one in 50,000 people worldwide. It affects men and women equally and can occur in all racial and ethnic groups. The exact incidence of HAE may be challenging to determine because the condition is often underdiagnosed or misdiagnosed.

The onset of HAE symptoms can occur at any age, but most people experience their first attack during childhood or adolescence. The symptoms of HAE can vary widely from person to person, including recurrent episodes of swelling in the face, airway, gastrointestinal tract, or genitals, as well as severe abdominal pain, nausea, and vomiting [2].

The lack of C1-INH is caused by the disorderly production of bradykinin (BK), a receptor that plays a significant role in vasopermeability [3]. The vascular permeability allows the receptor to help mitigate inflammation. When the BK receptor is uncontrolled by deficient or non-existent C1-INH receptors, it can cause swelling in the deep layer of the dermis of soft tissues. This often mirrors symptoms of a severe allergic reaction, where the patient presents with swollen eyes, face, lips, tongue, and other common areas such as extremities. Although it can often be mistaken as an allergic reaction, certain signs and symptoms and proper testing will allow for the correct diagnosis. For example, angioedema from HAE has a slower onset compared to histamine-induced angioedema and is usually unilateral; it is not pruritic and thus does not manifest as urticaria. HAE also has a greater incidence of abdominal symptoms [4]. Gastrointestinal tract involvement is common in HAE, with symptoms present in 33-50% of patients. These include nausea, vomiting, abdominal pain, and diarrhea secondary to intestinal edema [5]. A recent report details a case of bowel obstruction in an HAE patient; C1-INH treatment avoided the need for bowel resection [6].

When HAE is mistaken for histaminergic angioedema, unnecessary and ineffective treatment results. This is illustrated in the case report of a 14-year-old who was initially treated with diphenhydramine and methylprednisolone, only to find out she actually had HAE. This meant that the correct treatment was delayed while the patient suffered [7].

## Case presentation

**Figure 1:**
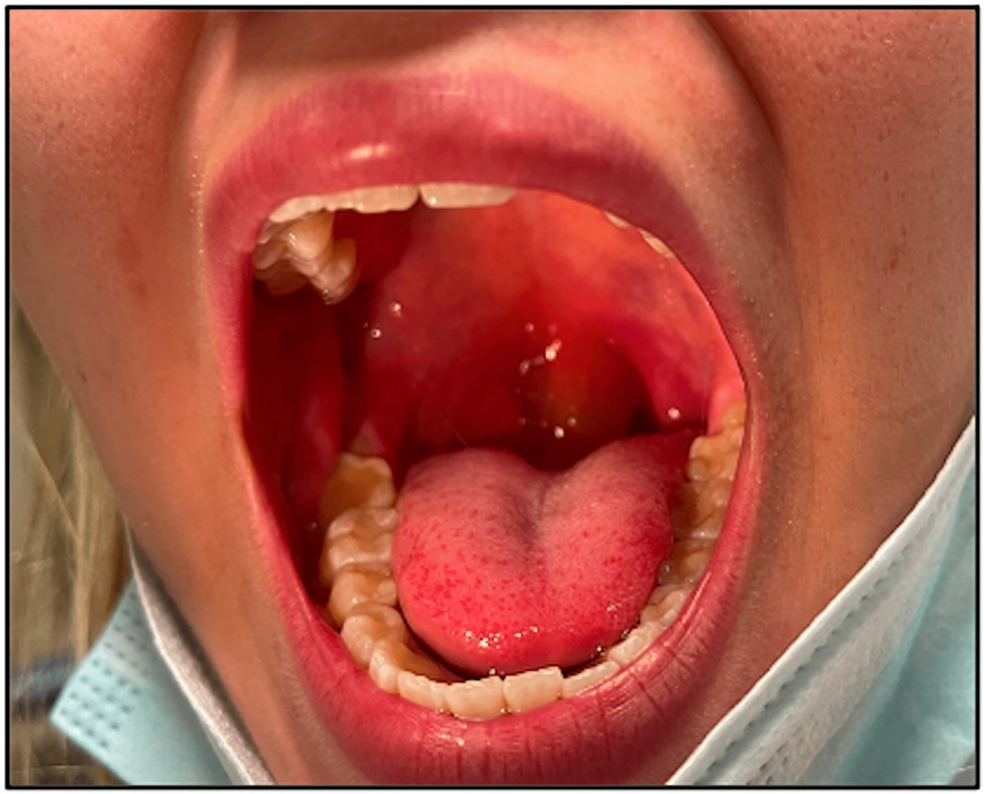
Clinical photograph depicting edematous uvula.

A 22-year-old female presented to the emergency room complaining of persistent sore throat and difficulty swallowing. The patient had no known allergies to report and denied any other potential causes for the severe swelling. She denied any shortness of breath or rash. The patient was aware of having HAE and arrived with her medication. Examination of the oropharynx revealed inflammation of the uvula (Figure 1).

The remainder of her physical exam was unremarkable, including a normal heart and lung exam. Her vital signs were as follows: blood pressure (BP) 120/73 mmHg, pulse rate 91 beats per minute, respiratory rate 19 breaths per minute, temperature 98.6°F, and room air saturation 98%.

She received an intravenous dose of 1000 units of C1 esterase inhibitor (Berinert®, CSL Behring, King of Prussia, Pennsylvania, United States) based on her weight of 50 kg. Following the administration, her symptoms improved. The patient was discharged home and advised to follow up with her primary care provider within three days.

## Discussion

There are three different types of HAE. HAE type I lacks the C1-inhibitor, type II has a defective C1-inhibitor, and type III has a normal C1-inhibitor [8]. Treatments target the production of BK to get it back into control, causing better functioning of the C1-INH [3]. 

Treatment of HAE consists of managing the spectrum of the condition. The first line of treatment is to attenuate the inflammation with C1 esterase replacement therapy. These medications work by inhibiting the formation or action of bradykinin. If there is any evidence of airway compromise, an emergent airway may be required. Prophylactically, HAE can be managed with regular C1-inhibitor replacement therapy (preferred) or an androgen such as danazol or stanozolol. Danazol is thought to work by promoting the synthesis of the C4 complement protein, resulting in a modest increase in the C1-INH level [9]. The use of anabolic steroids, such as danazol and stanozolol, has steadily decreased over the past decade in favor of more pathway-specific options, such as C1 esterase inhibitors [10].

Finally, as with any chronic disease, lifestyle changes are essential. For HAE, these include avoiding known triggers, such as certain infections, injuries, pain, trauma, menses, mental stress, and even weather changes [11].

HAE has several available treatments, all of which can be self-administered: pure plasma-derived C1-INH concentrate, icatibant acetate, ecallantide, and recombinant human C1-INH [9]. These medications all work to significantly reduce inflammation caused by malfunctions of the C1 inhibitor due to overproduction of the BK receptor. These medications are specifically formulated to reduce the production of BK, the receptor that causes angioedema. With the reduction of the BK receptor, the C1 inhibitors can function at a much more successful rate [3]. These medications can be given to help alleviate immediate symptoms and work well for short-term prevention.

Long-term therapy options include tranexamic acid, danazol, subcutaneous purified plasma, and lanadelumab [1]. Tranexamic acid inhibits the activation of plasminogen by plasmin and thus decreases bradykinin production. Lanadelumab is a human monoclonal antibody that targets plasma kallikrein [12]. These treatments can help the symptoms of HAE to the point where the disease can become a very minimal part of the patient's life. Though they can have outbreaks, knowing about these interventions and medications can help save many lives or people from prolonged pain. HAE attacks are, at times, extremely painful, sometimes even fatal. Angioedema resulting from HAE causes recurrent physical discomfort that can negatively impact the patient's participation in work and social activities [13]. Thus, it is crucial for patients to spot the signs of HAE and establish a plan to lessen the extremity and prevalence of swelling and irritation. 

## Conclusions

HAE can be a life-threatening condition if left untreated and can lead to severe complications such as airway obstruction, difficulty breathing, and severe abdominal pain. Early diagnosis and treatment of HAE are critical in order to prevent these complications and improve the quality of life for affected individuals.

## References

[1] Caballero T (2021). Treatment of hereditary angioedema. J Investig Allergol Clin Immunol.

[2] Serpa FS, Mansour E, Aun MV (2021). Hereditary angioedema: how to approach it at the emergency department?. Einstein (Sao Paulo).

[3] Kemp JG, Craig TJ (2009). Variability of prodromal signs and symptoms associated with hereditary angioedema attacks: a literature review. Allergy Asthma Proc.

[4] Hébert J, Boursiquot JN, Chapdelaine H (2022). Bradykinin-induced angioedema in the emergency department. Int J Emerg Med.

[5] Busse PJ, Christiansen SC (2020). Hereditary angioedema. N Engl J Med.

[6] Abdulkarim A, Craig TJ (2022). Hereditary angioedema. StatPearls [Internet].

[7] Tanaka A, Huh JY, Yamamoto T, Washio K, Ariyoshi K (2022). Bowel obstruction secondary to internal hernia in a hereditary angioedema patient: a case report. Int J Emerg Med.

[8] Lesser H, Cohn JE (2021). Hereditary angioedema. Int J Emerg Med.

[9] Fabiani JE, Paulin P, Simkin G, Leoni J, Palombarani S, Squiquera L (1989). Hereditary angioedema. Effect of danazol on C4 and functional C1INH (Article in Spanish). Rev Alerg Mex (1987).

[10] Anderson J, Maina N (2022). Reviewing clinical considerations and guideline recommendations of C1 inhibitor prophylaxis for hereditary angioedema. Clin Transl Allergy.

[11] Savarese L, Mormile I, Bova M, Petraroli A, Maiello A, Spadaro G, Freda MF (2021). Psychology and hereditary angioedema: a systematic review. Allergy Asthma Proc.

[12] Kenniston JA, Faucette RR, Martik D (2014). Inhibition of plasma kallikrein by a highly specific active site blocking antibody. J Biol Chem.

[13] West JB, Poarch K, Lumry WR (2021). Preventive treatment of hereditary angioedema: a review of phase III clinical trial data for subcutaneous C1 inhibitor and relevance for patient management. Clin Ther.

